# Regeneration of Cartilage in Human Knee Osteoarthritis with Autologous Adipose Tissue-Derived Stem Cells and Autologous Extracellular Matrix

**DOI:** 10.1089/biores.2016.0024

**Published:** 2016-08-01

**Authors:** Jaewoo Pak, Jung Hun Lee, Kwang Seung Park, Byeong Chul Jeong, Sang Hee Lee

**Affiliations:** ^1^ Stems Medical Clinic, Seoul, Republic of Korea.; ^2^ TEDA-Puhua International Hospital, Tianjin, China.; ^3^ Life Science Institute, Komplek Permata Senayan, Jalan Tentara Pelajar, Jakarta Selatan, Indonesia.; ^4^ National Leading Research Laboratory, Department of Biological Sciences, Myongji University, Yongin, Republic of Korea.

**Keywords:** adipose tissue-derived stem cells, extracellular matrix, human cartilage regeneration, osteoarthritis

## Abstract

This clinical case series demonstrates that percutaneous injections of autologous adipose tissue-derived stem cells (ADSCs) and homogenized extracellular matrix (ECM) in the form of adipose stromal vascular fraction (SVF), along with hyaluronic acid (HA) and platelet-rich plasma (PRP) activated by calcium chloride, could regenerate cartilage-like tissue in human knee osteoarthritis (OA) patients. Autologous lipoaspirates were obtained from adipose tissue of the abdominal origin. Afterward, the lipoaspirates were minced to homogenize the ECM. These homogenized lipoaspirates were then mixed with collagenase and incubated. The resulting mixture of ADSCs and ECM in the form of SVF was injected, along with HA and PRP activated by calcium chloride, into knees of three Korean patients with OA. The same affected knees were reinjected weekly with additional PRP activated by calcium chloride for 3 weeks. Pretreatment and post-treatment magnetic resonance imaging (MRI) data, functional rating index, range of motion (ROM), and pain score data were then analyzed. All patients' MRI data showed cartilage-like tissue regeneration. Along with MRI evidence, the measured physical therapy outcomes in terms of ROM, subjective pain, and functional status were all improved. This study demonstrates that percutaneous injection of ADSCs with ECM contained in autologous adipose SVF, in conjunction with HA and PRP activated by calcium chloride, is a safe and potentially effective minimally invasive therapy for OA of human knees.

## Introduction

Adipose tissue is an excellent source of mesenchymal stem cells (MSCs) and can be easily obtained by performing liposuction. To obtain stem cells, this liposuctioned adipose tissue, termed lipoaspirates, is usually incubated with collagenase for a short time period at human body temperature. Since adipose tissue mainly comprises adipocytes and extracellular matrix (ECM), which contains various blood vessels, the end product obtained by incubating the lipoaspirates with collagenase is called stromal vascular fraction (SVF).^1^ These adipose SVFs have shown to contain various components, including MSCs, RBCs, WBCs, and fibrous tissue of the ECM.^1^ These MSCs obtained from adipose tissue, termed adipose tissue-derived stem cells (ADSCs or ASCs), have shown to regenerate cartilage *in vitro*.^2,3^ In addition, it has been well recognized that ECM may work as a scaffold and may enhance stem cell adherence to the cartilage lesion,^4^ and ECM has been shown to excrete various growth factors, which may also enhance the survival and growth of stem cells injected.^4^ Thus, autologous adipose SVF containing both stem cells and ECM can potentially be an excellent agent for cartilage regeneration.

Currently, there are numerous ways to process autologous adipose tissue to obtain SVF. Although most of these methods involve incubating adipose tissue with collagenase, some subtle differences exist. The differences may lie in types and amount of collagenase, time duration for the incubation, temperatures of incubation, forces and time of centrifuge to remove the leftover collagenase, etc. Such differences in processing autologous adipose tissue for generating adipose SVF may influence its regeneration capability, causing variable results. The differences in the process may affect the quantity and quality of ADSCs and ECM generated. Different quantity and quality of ECM may have direct effects on stem cell viability, adherence, and growth. In addition, all these methods do not involve homogenization of the lipoaspirates and thus collagenases may have uneven effects: small-sized lipoaspirates are well digested and the large ones are not. Such uneven breakdown of the matrix of the lipoaspirates can result in large-sized fibrous tissue as well as small. The larger sized fibrous tissue may work fine as a volume expander; however, when injected in a joint, it may cause unknown responses and results. These large-sized fibrous matrix tissues can also clog up the syringes and needles used for joint injection as well, necessitating needle changes and thus increasing the possibility of bacterial introduction to the joint. In addition to the lack of standardization, there also is a very large individual variation in the content of adipose SVF.^5^ However, to the best of our knowledge, none of the methods reported thus far involve homogenizing the autologous adipose tissue for human patients.

Adipose tissue SVF has been widely used in Korea over decades by plastic surgeons as a semipermanent volume expander. In June 2009, the Korean Food and Drug Administration (KFDA) had allowed adipose SVF to be used as a medical procedure when obtained and processed within a same medical facility with minimal processing.^6^ Afterward, autologous adipose SVF has been applied successfully in the field of orthopedics as a potential agent to regenerate cartilage in human patients in Korea. In 2011, Pak showed that ADSCs contained in the adipose SVF, injected with platelet-rich plasma (PRP), can regenerate cartilage-like tissue in human osteoarthritis (OA) patients.^7^ Many other studies have confirmed that autologous ADSCs in human adipose SVF are potential agents capable of regenerating cartilage in OA patients.^8^

In this study, in an effort to minimize the variability of the contents of autologous adipose SVF and also to reduce the potential unknown side effects of the large-sized fibrous tissue, the lipoaspirates were minced to homogenize the ECM. With homogenization of the lipoaspirates, collagenase may have a more uniform effect in breaking down the matrix when incubated, theoretically releasing stem cells more evenly than nonhomogenized adipose tissue. In addition, with homogenization, the fibrous tissue of the ECM is cut into smaller pieces, preventing the clogging problem.

In this report, the PRP was used as a source of growth factors and as a differentiating agent for the ADSCs injected. PRP contains various growth factors, which have been shown to have positive effects on growth and differentiation of various stem cells to chondrocyte formation.^9–11^ Hyaluronic acid (HA) was used as scaffolding and to enhance stem cell penetration of the cartilage matrix.^4,12^ Furthermore, we present, for the first time in our knowledge, the positive effects of autologous lipoaspirates containing both ADSCs and homogenized ECM in improving the pain in terms of visual analog scale (VAS) and function, in terms of physical therapy parameters, along with magnetic resonance imaging (MRI) evidence of cartilage-like tissue regeneration in human knee OA patients after 3 months of treatment.

## Materials and Methods

The new rules and regulations by the KFDA have made it possible to use autologous adipose tissue as a source of ADSCs in Korea. At the time, an informed consent was also obtained from the patient. The approval and consent to report single cases were waived by the Myongji University Institutional Review Board committee (MJUIRB) for case report. Furthermore, this clinical study was in compliance with the Declaration of Helsinki and regulation guidelines of the KFDA.

### Inclusion and exclusion criteria and outcome end-points

The inclusion criteria, exclusion criteria, and outcome end-points are listed as follows. Inclusion criteria: MRI evidence of the hip OA; orthopedic evaluation with negative Apley and McMurray tests that determined that the patient was a candidate for a total knee replacement (TKR) surgery; males or females; 50 years of age or over; unwillingness to proceed with TKR; failure of conservative management; and ongoing disabling pain. Exclusion criteria: active inflammatory or connective tissue disease thought to impact pain condition (i.e., lupus, rheumatoid arthritis, fibromyalgia); active endocrine disorder that might impact pain condition (i.e., hypothyroidism, diabetes); active neurologic disorder that might impact pain condition (i.e., peripheral neuropathy, multiple sclerosis); active cardiac disease; and active pulmonary disease requiring medication usage. Outcome end-points: pre- and post-treatment VAS, functional rating index (FRI), range of motion (ROM), and MRI before the treatment and 3 months after the treatment.

Three patients, 87-year-old female with stage 3 OA, 68-year-old male with stage 3 OA, and 60-year-old female with stage 3 OA, were included. They all had no significant past medical history. After taking MRI studies, these patients were treated with autologous adipose SVF with calcium chloride-activated autologous PRP and HA at day 0. Subsequently, the patients returned to the clinic every week × 3 for HA and autologous PRP activated with calcium chloride. The patients were assessed at the weeks of 2, 4, and 16 (18 or 22) for pain improvement in terms of VAS and function improvement in terms of physical therapy parameters. In addition, these patients were followed by post-treatment MRI 3 months after the treatment.

### Pain score and physical therapy

FRI, VAS, and ROM were determined as previously described.^13,14^ Apley and McMurray tests were performed on all patients on initial physical examinations.

### Medication restrictions

Patients were restricted from taking steroids, aspirin, nonsteroidal anti-inflammatory drugs (NSAIDs), and Asian herbal medications for 1 week before the procedure.

### Liposuction and preparation of ADSC/ECM mixture

In the operating room, ∼50 mL of packed adipose tissue was obtained by liposuction of the subcutaneous layer of the lower abdominal area using manual techniques.^7^ In brief, after cleaning the lower abdominal area with betadine, the patient was draped using the sterile technique. Using tumescent solution, the lower abdomen was anesthetized. Afterward, using 3.0 cannula connected to a 60-mL Luer-Lock syringe, ∼100 g of adipose tissue was obtained. These lipoaspirates were then transferred to centrifuge syringe barrel (CPL™; Medicamatch, Ansan, Korea) and centrifuged at 1600 *g* for 5 min. The packed adipose tissue was then transferred back to the 60-mL Luer-Lock syringe that was connected to another 60-mL Luer-Lock syringe through manual tissue homogenizer that contains blades. The lipoaspirates were then pushed to the other 60-mL Luer-Lock syringe through the homogenizer for 40 times, resulting in cutting and mincing of the adipose tissue and filtering large fibrous tissue. These minced lipoaspirates were then transferred back to the 60-mL centrifuge syringes and mixed thoroughly with collagenase (0.07% type 1 collagenase; Adilase; Worthington, Lakewood, NJ). The centrifuge syringe with the mixture was then incubated in a rotating incubator mixer at 37°C for 40 min. After the incubation, the SVF and collagenase mixture in the centrifuge syringe was centrifuged at 300 *g* to separate and remove collagenase,^2,3,7^ and the top part of the solution was removed and discarded. Then, the 60-mL centrifuge syringe was filled with dextrose 5% in the normal saline solution (D5NS; Baxter Healthcare Corp., Marion, NC) upto 50 mL and centrifuged again. This process was repeated for a total of three times. After the last centrifuge, the total volume of the SVF containing both ADSCs and ECM along with other cells and tissue obtained was 7.5–8.5 mL.

### PRP preparation

While preparing the ADSCs and ECM, 30 mL autologous blood was drawn along with 2.5 mL anticoagulant citrate dextrose solution (0.8% citric acid, 0.22% sodium citrate, and 0.223% dextrose; Baxter Healthcare Corp.). After centrifugation (300 *g*, 15 min, and then 1200 *g*, 5 min), 4.4 mL of PRP was obtained and 3% (w/v) calcium chloride (0.1 mL; Choongwae Pharmaceutical Co., Gyeonggido, Korea) was added to the mixture to activate PRP; 0.5% (w/v) HA (2 mL; Huons, Chungbuk, Korea) was added as a scaffold to this mixture. These ADSCs and ECM, along with PRP activated by calcium chloride and HA, stand for the ADSC/ECM mixture.

### ADSC/ECM mixture-based treatment

After cleaning the knee with 5% povidone–iodine (Choongwae Pharmaceutical Co., Seoul, Korea) and draping it in a sterile manner, the injection site was anesthetized with 0.25% ropivacaine (Huons) superficially outside of joint capsule and diluted 0.125% lidocaine (Daehan Pharmaceutical Co., Gyeonggido, Korea) was used to anesthetize inside the joint capsule. On the same day of liposuction, the ADSC/ECM with PRP and HA mixture (∼14 mL) was injected into the medial inferior tibiofemoral joint of the knees using a 38-mm 18-gauge needle under ultrasound guidance. The patient was then instructed to remain still for 60 min to allow for cell attachment. As the patient was discharged from the clinic, the patient was instructed to limit activities to minimal for 1 week. The patient returned for three additional injections of PRP activated by calcium chloride over 3 weeks.

### Statistical analysis

Sample sizes for outcomes of pain measurements and ROM were *n* = 3. Bar graphs are presented as the mean ± standard error of the mean (SEM) with statistically significant differences defined as *p* < 0.05 using two-way analysis of variance with Bonferroni *post hoc* tests for multiple comparisons.

## Results

### Patient case #1

The patient is an 87-year-old Korean female with more than 20-year history of bilateral knee pain. The left knee pain is worse than the right. Even with chronic knee pain, the patient was leading a relatively active life style until recently when her knee pain progressed in severity and started to limit her daily activities.

**FIG. 1.**
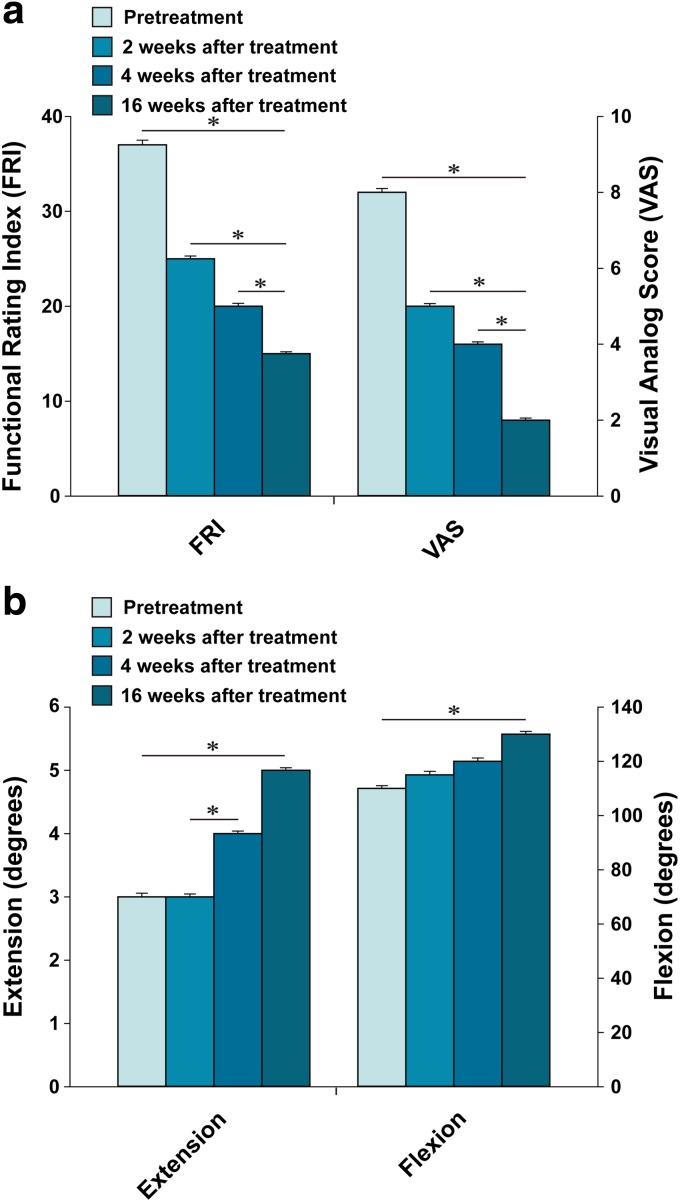
Outcome of pain measurements **(a)** and range of motion **(b)** from patient #1. *Indicates a statistically significant finding (*p* < 0.05).

**FIG. 2.**
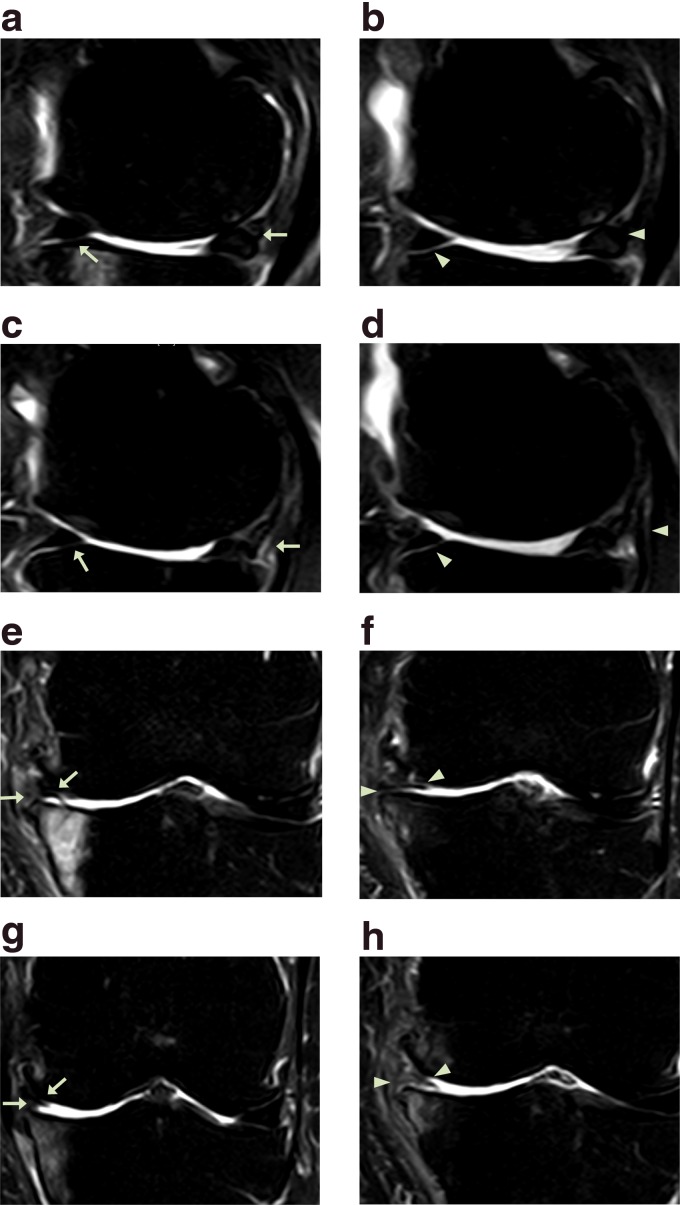
MRI sagittal **(a–d)** and coronal **(e–h)** sequential T2 views of the knee from patient #1. Pretreatment MRI scans **(a**: sequential image, 5/20; **c**: 6/20; **e**: 10/20; and **g**: 11/20**)** show cartilage lesions (arrows). Post-treatment MRI scans at 16 weeks **(b**: 6/20; **d**: 7/20; **f**: 10/20; and **h**: 11/20**)** indicate cartilage-like tissue regeneration (arrowhead) that has been repaired by ADSC/ECM mixture-based treatment. ADSC, adipose tissue-derived stem cell; MRI, magnetic resonance imaging.

With the diagnosis of stage 3 OA of the knee, the patient had received multiple injections of steroids and HA over the last couple of years. However, she did not notice any permanent improvement of the pain. The patient's knee condition further deteriorated. Right before the initial office visit, the patient was offered TKR surgery by an orthopedic surgeon. However, she was reluctant to have the surgery due to possible side effects. At the time of initial evaluation, the patient reported severe pain (VAS score of 8; Fig. 1a) on rest. The patient also complained of increased pain (FRI: 37; Fig. 1a) when walking up and down stairs. On physical examination, there was mild joint edema, decreased ROM, and tenderness with flexion (Fig. 1b). Apley's and McMurray's tests were negative, and there was no ligament laxity. A pretreatment MRI demonstrated a decreased size and deformed contour on medial meniscus of the left knee due to maceration (Fig. 2a, c, e, g).

This patient received the treatment as described in the Materials and Methods section. After the second week of ADSC/ECM mixture injection, the patient's pain and ROM improved (Fig. 1a, b). By the 16th week, the patient's pain and ROM significantly improved more than 70% (Fig. 1a, b). Post-treatment MRI taken after the 16th week showed increased thickness of cartilage-like tissue on the medial side of the knee (Fig. 2b, d, f, h).

### Patient case #2

**FIG. 3.**
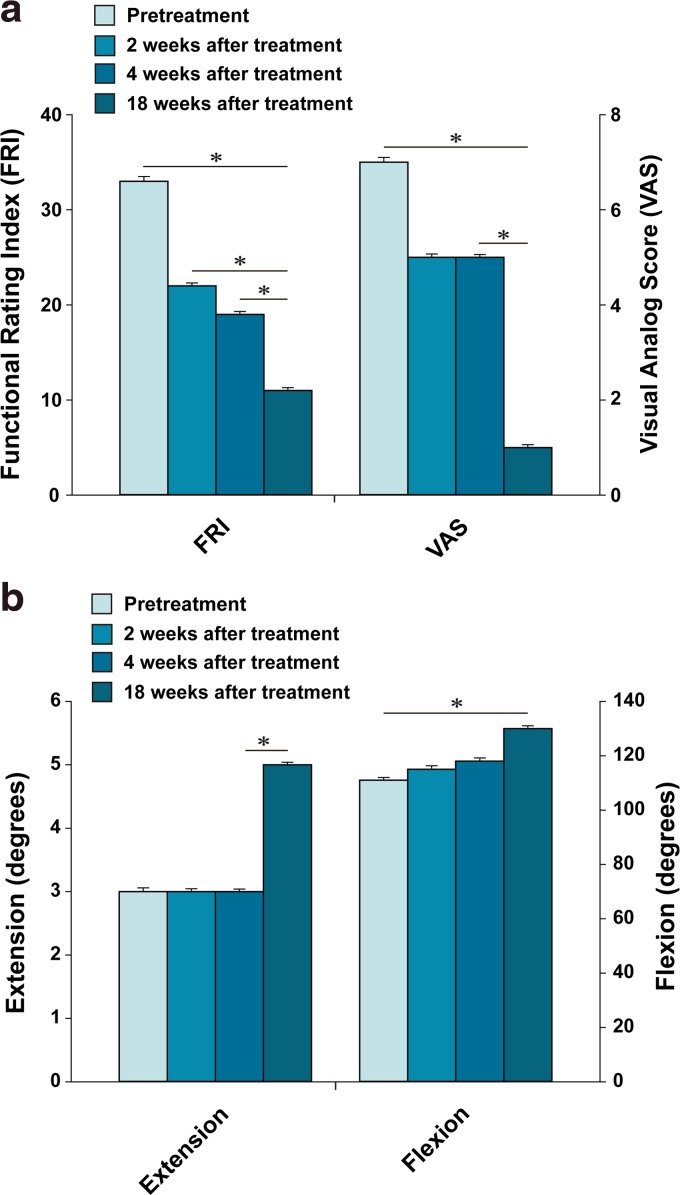
Outcome of pain measurements **(a)** and range of motion **(b)** from patient #2. *Indicates a statistically significant finding (*p* < 0.05).

**FIG. 4.**
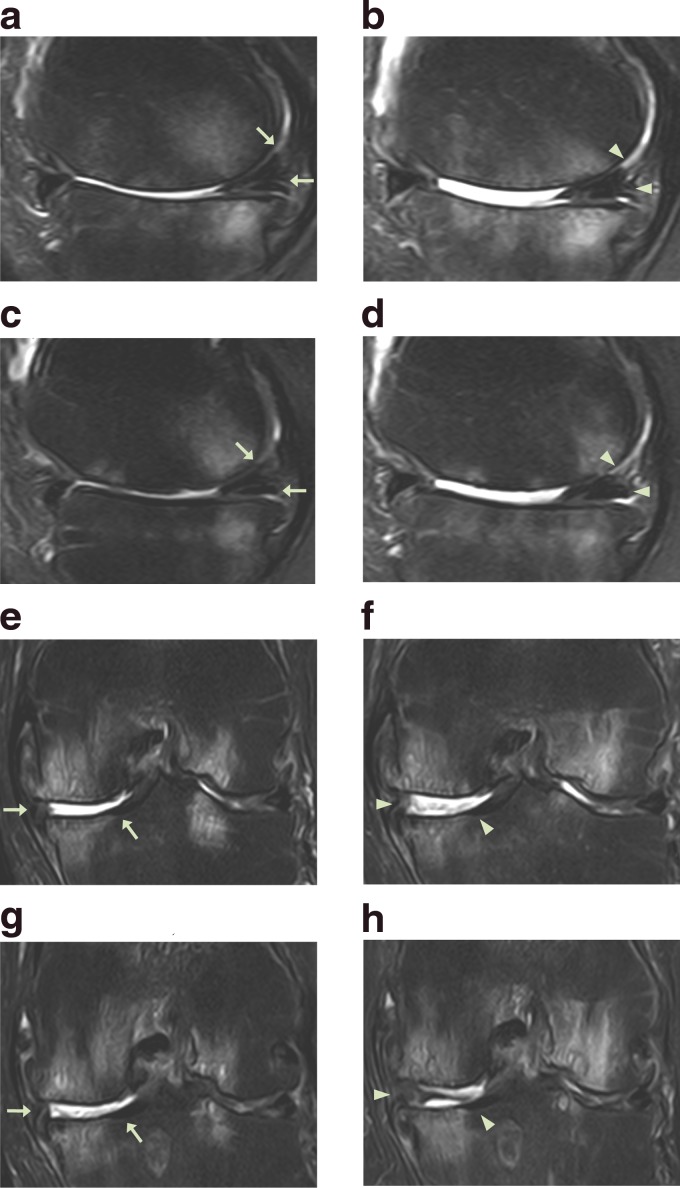
MRI sagittal **(a–d)** and coronal **(e–h)** sequential T2 views of the knee from patient #2. Pretreatment MRI scans **(a**: sequential image, 6/20; **c**: 7/20; **e**: 13/20; and **g**: 14/20**)** show cartilage lesions (arrows). Post-treatment MRI scans at 18 weeks **(b**: 6/20; **d**: 7/20; **f**: 13/20; and **h**: 14/20**)** indicate cartilage-like tissue regeneration (arrowhead) that has been repaired by ADSC/ECM mixture-based treatment.

The patient is a 68-year-old Korean male with more than 2-year history of left knee pain. Initially, 2 years before the office visit, the patient was diagnosed with having OA of knee with meniscus tear. The patient subsequently received left medial meniscus resection and debridement through arthroscopy. However, after arthroscopic meniscectomy and debridement, the patient's knee pain did not improve. Furthermore, multiple injections of steroid and HA did not provide any improvement. The patient was seen by another orthopedic surgeon who offered the patient TKR surgery. The patient was reluctant to go through TKR surgery due to potential side effects. At the time of initial evaluation, the patient reported severe pain on the left knee (VAS score: 7; Fig. 3a) on rest. The pain (FRI: 33; Fig. 3a) increased when walking up and down stairs. On physical examination, there was mild deformity with mild joint swelling. ROM (Fig. 3b) was decreased. Apley's and McMurray's tests were negative, and there was no ligament laxity. A pretreatment MRI demonstrated a decreased size and deformed contour on medial meniscus due to the meniscectomy of the left knee along with cartilage thinning (Fig. 4a, c, e, g) and the patient was diagnosed as having stage 3 OA.

The patient went through the identical procedure as patient case #1. After the second week of ADSC/ECM mixture injection, the patient's pain and ROM improved (Fig. 3a, b). By the 18th week, the pain and ROM significantly improved to 80% (Fig. 3a, b). Repeated MRI taken after the 18th week showed an increase in the height of cartilage-like tissue on the anterior medial side of the knee (Fig. 4b, d, f, h).

### Patient case #3

**FIG. 5.**
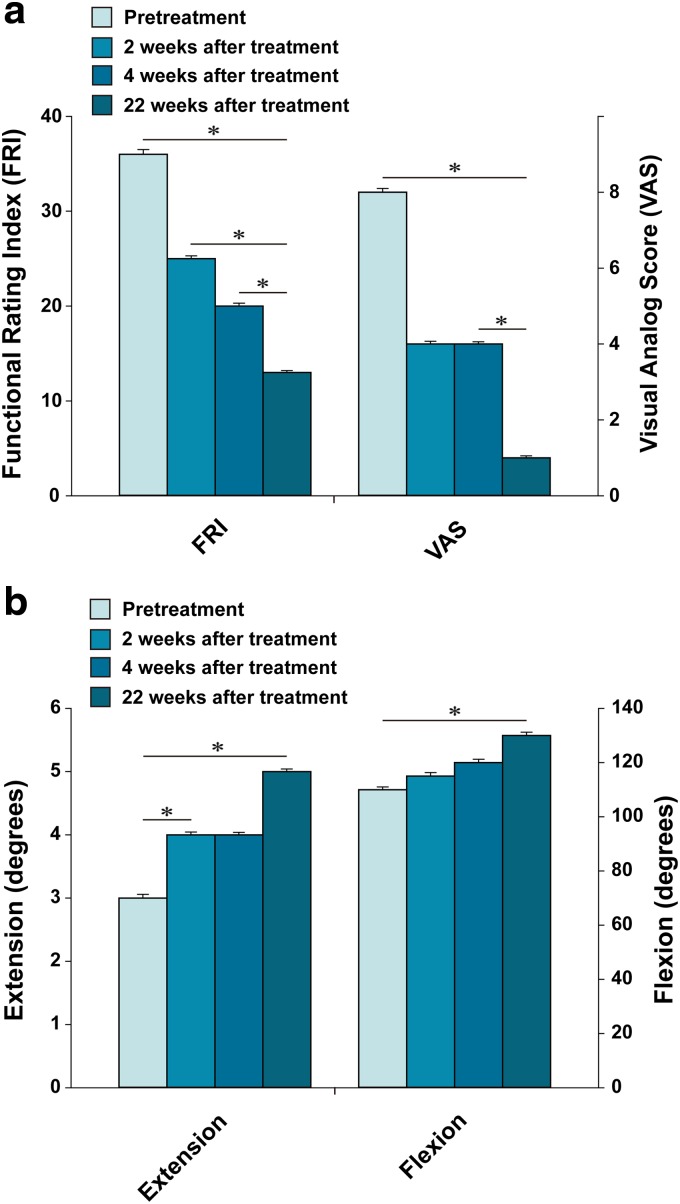
Outcome of pain measurements **(a)** and range of motion **(b)** from patient #3. *Indicates a statistically significant finding (*p* < 0.05).

**FIG. 6.**
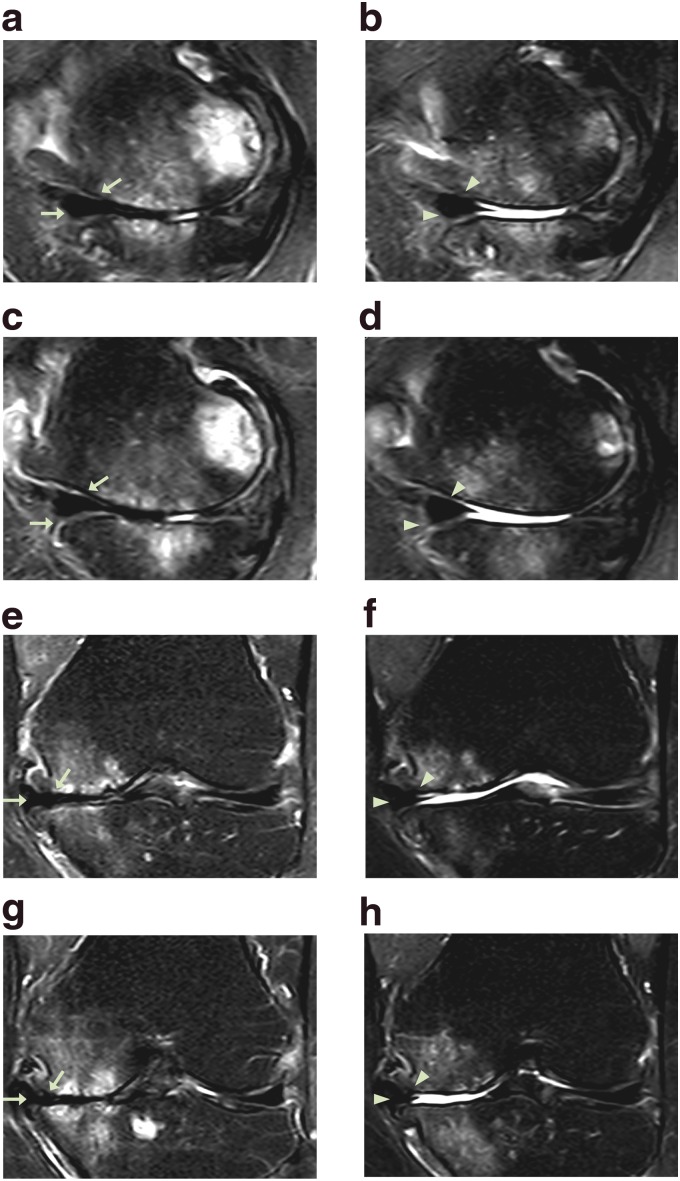
MRI sagittal **(a–d)** and coronal **(e–h)** sequential T2 views of the knee from patient #3. Pretreatment MRI scans **(a**: sequential image, 4/20; **c**: 5/20; **e**: 10/20; and **g**: 11/20**)** show cartilage lesions (arrows). Post-treatment MRI scans at 22 weeks **(b**: 4/20; **d**: 5/20; **f**: 10/20; and **h**: 11/20**)** indicate cartilage-like tissue regeneration (arrowhead) that has been repaired by ADSC/ECM mixture-based treatment.

The patient is a 60-year-old Korean female with more than 8-year history of left knee pain due to stage 3 OA. The patient had received an arthroscopic lavage/debridement and multiple injections of steroids and HA without any prolonged improvement. This patient was also offered TKR by an orthopedic surgeon. However, the patient was reluctant to go through TKR surgery due to potential side effects. At the time of initial evaluation, the patient reported severe pain (VAS score: 8; Fig. 5a) on rest. The pain (FRI: 36; Fig. 5a) increased when walking. The patient also complained of mild knee swelling. ROM (Fig. 5b) was decreased. Apley's and McMurray's tests were negative, and there was no ligament laxity. A pretreatment MRI demonstrated a decreased size and deformed contour on medial meniscus of the left knee due to maceration along with cartilage thinning (Fig. 6a, c, e, g).

Afterward, this patient went through the same procedure as patient case #1 and #2. After the second week of ADSC/ECM mixture injection, the patient's pain and ROM improved very much, ∼50% (Fig. 5a, b). By the 22th week, the pain and ROM significantly improved over 80% (Fig. 5a, b). Repeated MRI taken after the 22nd week showed an increase in the height of the cartilage-like tissue on the medial side of the knee (Fig. 6b, d, f, h).

## Discussion

ADSCs exist within ECM.^2,3^ To obtain autologous ADSCs, it has become customary for adipose tissue to be digested with collagenase to breakdown the collagen component of ECM. Consequently, the end result of adipose SVF contains different sizes of collagen fibers along with other cells and tissue. Furthermore, it has been shown that ECM may play an important role in the field of regenerative medicine. ECM has shown to provide various growth factors and work as a scaffold for cells to attach and grow.^4^ Thus, this autologous adipose SVF with ECM has been used safely in the last few years in the field of orthopedics.^8,15^

However, large-sized fibers of the ECM can be generated by uneven penetration of collagenase into different sizes of lipoaspirates. These large-sized fibers may clog up the syringes and needles, necessitating multiple needle changes when injecting them. Frequent needle changes when performing joint injections may increase the chance of bacterial introduction to the joint. Furthermore, these large-sized fibers, when introduced into a joint, may cause unknown results, responses, and side effects.

ECM is an inherent part of adipose tissue and has been used safely in the field of orthopedics as a part of autologous adipose SVF with occasional clogging of the syringes and needles by large-sized fibrous tissue of ECM. Thus, to decrease the size of the fiber contents of ECM, the lipoaspirates were minced and homogenized. In such a way, no large fibrous content of the ECM would be injected into a joint and no clogging problems would arise. In addition, minced and homogenized ECM may enhance the regenerative effect of autologous ADSCs. This clinical case series provides for the first time that minced and homogenized autologous adipose tissue SVF can be used safely and effectively in potentially regenerating cartilage in human OA patients, evidenced by clear MRI results of cartilage-like tissue regeneration.

Based on MRI features, it is probable that the newly formed tissue is cartilage in nature in all three patients, although the true nature of the newly formed tissue cannot be confirmed without a biopsy. While cartilage regeneration in human knees using ADSCs in the form of SVF has been shown in numerous human patients, this case series is the very first study suggesting possibly a safer strategy of using autologous adipose SVF for cartilage in human knees. In addition to the MRI evidence, the patients' symptoms and signs improved as in earlier times than previously reported by Pak in 2011.^7^ All three patients reported symptom improvement by the second week of treatment. This can be attributed to the fact that the lipoaspirates were minced and homogenized. However, the true effects of homogenized ECM in the joint need to be further studied and clarified.

As shown previously, none of the patients reported 100% resolution of their symptoms. This may be due to the fact that OA is a disease of the whole joint, not just cartilage. Thus, with improved cartilage regeneration, the patients may need to further strengthen the related tendons, ligaments, and muscles to further improve already improved joint conditions.

Cartilage regeneration with adipose SVF depends on numerous factors: number of stem cells and the viability, adherence, growth, and differentiation of ADSCs injected. Collagenase probably is one of the important factors that affect the total yield of the number of stem cells in the SVF. Too high concentration of collagenase may have detrimental effects to the stem cell viability and too less may not be as effective in releasing stem cells from the matrix.^16^ In addition, standardization of the collagenase is further complicated by individual patient variations. The number of stem cells that can be obtained from each individual patient varies greatly.^5^ Aging and the degree of obesity, for example, may affect the texture of subcutaneous tissue of each individual patient. Thus, collagenase may have different effects on each individual patient's adipose tissue,^17^ causing variation in the number of ADSCs in the autologous adipose SVF. Since high concentration of collagenase may be detrimental to viability of stem cells, it would be a better strategy to use less potent collagenase and use homogenizer. With homogenizer, the fat tissue is cut into small pieces for better even penetration of the collagenase, and some of the large fibrous tissue is filtered as well. With better even penetration of collagenase, it is possible to obtain more stem cells and more uniformed homogenized ECM, which may have further benefits in cartilage regeneration. However, more studies are needed to confirm this theory.

Autologous PRP was used as a source of growth factors and differentiating factors for ADSCs. PRP contains various growth factors. These growth factors have shown to stimulate growth of stem cells as well as differentiation of stem cells to chondrocytes.^11^ Although HA was used as additional scaffolds for stem cells to attach to the lesion, symptom improvement can also be attributed to the HA injected.

Although further studies are needed to verify this procedure presented in this report, this is the very first case series attempting to improve the current strategy of using autologous adipose SVF in human knee OA with percutaneous injections. Currently, no nonsurgical curative therapy is available for the treatment of OA. However, percutaneous injection of stem cell therapy with ADSCs and homogenized ECM along with HA and autologous PRP activated with calcium chloride may provide an alternative to current treatment strategy of treating knee OA.

## Conclusions

Autologous adipose SVF containing both ADSCs and homogenized ECM is a new potential strategy to treat OA of human knees. With percutaneous injections of autologous adipose ADSCs and homogenized ECM with HA and calcium chloride-activated autologous PRP, patients' symptoms improved in knees, maybe faster than nonhomogenized ECM. All clinical criteria of FRI, VAS score, and ROM improved in all patients, along with significant MRI changes. Although no biopsy of the regenerated tissue was performed, the tissue can be estimated to be cartilage-like in nature by comparing with the surrounding cartilage and other tissues. Although further studies are necessary to verify this new procedure, percutaneous injections of autologous adipose SVF containing ADSCs and homogenized ECM along with HA and autologous PRP activated with calcium chloride present a promising minimally invasive option of treating OA of human knees by regenerating cartilage-like tissue.

## Abbreviations Used

ADSC: autologous adipose tissue-derived stem cell
ECM: extracellular matrix
FRI: functional rating index
HA: hyaluronic acid
MRI: magnetic resonance imaging
MSC: mesenchymal stem cell
OA: osteoarthritis
PRP: platelet-rich plasma
ROM: range of motion
SVF: stromal vascular fraction
TKR: total knee replacement
VAS: visual analog scale
